# Genetic Analysis and QTL Mapping of Seed Coat Color in Sesame (*Sesamum indicum* L.)

**DOI:** 10.1371/journal.pone.0063898

**Published:** 2013-05-21

**Authors:** Haiyang Zhang, Hongmei Miao, Libin Wei, Chun Li, Ruihong Zhao, Cuiying Wang

**Affiliations:** Henan Sesame Research Center, Henan Academy of Agricultural Sciences, Zhengzhou, Henan, China; Pennsylvania State University, United States of America

## Abstract

Seed coat color is an important agronomic trait in sesame, as it is associated with seed biochemical properties, antioxidant content and activity and even disease resistance of sesame. Here, using a high-density linkage map, we analyzed genetic segregation and quantitative trait loci (QTL) for sesame seed coat color in six generations (P_1_, P_2_, F_1_, BC_1_, BC_2_ and F_2_). Results showed that two major genes with additive-dominant-epistatic effects and polygenes with additive-dominant-epistatic effects were responsible for controlling the seed coat color trait. Average heritability of the major genes in the BC_1_, BC_2_ and F_2_ populations was 89.30%, 24.00%, and 91.11% respectively, while the heritability of polygenes was low in the BC_1_ (5.43%), in BC_2_ (0.00%) and in F_2_ (0.89%) populations. A high-density map was constructed using 724 polymorphic markers. 653 SSR, AFLP and RSAMPL loci were anchored in 14 linkage groups (LG) spanning a total of 1,216.00 cM. The average length of each LG was 86.86 cM and the marker density was 1.86 cM per marker interval. Four QTLs for seed coat color, QTL1-1, QTL11-1, QTL11-2 and QTL13-1, whose heritability ranged from 59.33%–69.89%, were detected in F_3_ populations using CIM and MCIM methods. Alleles at all QTLs from the black-seeded parent tended to increase the seed coat color. Results from QTLs mapping and classical genetic analysis among the P_1_, P_2_, F_1_, BC_1_, BC_2_ and F_2_ populations were comparatively consistent. This first QTL analysis and high-density genetic linkage map for sesame provided a good foundation for further research on sesame genetics and molecular marker-assisted selection (MAS).

## Introduction

Sesame (*Sesamum indicum* L., 2n = 26), a member of the Pedaliaceae family, is one of the oldest and most important oilseed crops known to man due to its high oil content and quality [1]. Sesame seed is also rich in proteins, vitamins, niacin, minerals and lignans and is popularly used as a food and medicine [2]–[3]. Seed coat color is an important agronomic trait in sesame. The natural color of mature sesame seeds varies from black, intermediate colors (e.g., gray, brown, golden, yellow and light white) to white. Compared with white seeds, black sesame seeds usually have higher ash and carbohydrate content, but lower protein, oil, and moisture ratios [4]. In East Asia, the products of black sesame seeds attract greater acceptance. Seed coat color in sesame seems to be associated with seed biochemical properties, antioxidant content and activity and even the level of disease resistance among sesame accessions, in addition to being a marker of evolution within the *Sesamum* genus [2]–[7].

Significant attention has been paid to the inheritance of sesame seed coat color over a long period. The complex nature of the seed coat color trait had been mentioned in many reports. Nohara et al. (1933) performed a cross between white-seeded and black-seeded sesame accessions, obtaining an F_2_ ratio of 9∶3∶3∶1 for black, dark brown, pale brown, and white seed coat colors, respectively, and concluded that seed coat color trait is regulated by two genes [8]. In another crosses between black and dirty white sesame seed types, black was invariably dominant in the F_1_ generation, and an F_2_ segregation ratio of 9 black: 3 grey: 4 dirty white was obtained. Results also suggested that seed coat color is regulated by two genes [9]. Gutierrez et al. (1994) found that black is the dominant testa color and light brown was observed to be partially dominant over white. They concluded that coat color is controlled by two independent genes with complementary effects and complete dominance at each locus [10]. However, examination of the F_2_ generation in black × light brown and black × white crosses revealed that one gene had complete dominance and supplemented the effects of other genes controlling basic testa colors [10]. Baydar and Turgut (2000) reported that epistatic segregation (9∶4∶3 and 9∶3∶4 ratios) determines sesame seed coat color [11]. In addition, a recent analysis of crosses between nine sesame accessions from Nigeria also demonstrated that seed color has a complex genetic basis, accessions with the same seed coat color possibly having different genotypes [12].

Genetic segregation analysis over multi-generations and mapping of quantitative trait loci (QTL) are the main approaches taken to clarify the genetic basis of quantitative traits [13]–[16]. To date, there are no reports on QTL mapping for sesame traits due to the lack of a high-density linkage map. Recent progresses on sesame genetic mapping and the development of molecular markers has laid an important foundation for studies on the genetics and QTL analysis of important sesame traits [17]–[18].

The aims of this study were to (1) comprehensively analyze segregation over multiple generations (P_1_, P_2_, F_1_, F_2_, BC_1_ and BC_2_) of a cross between black and white sesame accessions, and to explain the inheritance of sesame seed color trait, (2) construct a high-density genetic linkage map with a mapping population of 260 F_2_ and F_3_ progenies, and (3) locate the first sesame QTL for seed coat color on the linkage map.

## Materials and Methods

### Plant Materials

**Figure 1:**
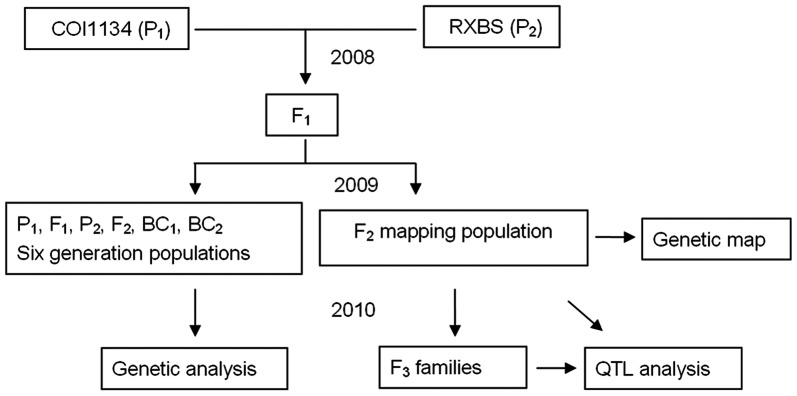
Schematic diagram of the sesame populations used for seed coat color trait analysis. Populations derived from six generations of P_1_, F_1_, P_2_, F_2_, BC_1_, BC_2_, were investigated to determine the genetic basis of the seed coat color trait. 260 lines of an F_2_ mapping population were screened to construct a genetic linkage map. 260 lines of an F_2_ mapping population and their F3 families were used for QTL determination.

The two *S. indicum* germplasm samples used, COI1134 (white seeded, P_1_) and RXBS (black seeded, P_2_) were accessions from the sesame germplasm resources collection at Henan Sesame Research Center, Henan Academy of Agricultural Sciences (HAAS). To investigate segregation, three replicates of the P_1_, P_2_, F_1_, BC_1_, BC_2_ and F_2_ populations were grown at Yuanyang experimental station, HAAS, in 2009 (Figure 1). To construct a genetic linkage map and locate QTLs, an F_2_ population of 260 lines was grown at Yuanyang experimental station and the corresponding 260 F_3_ families were grown at both Yuanyang and Pingyu experimental stations in 2010. Young leaf tissues from parents and F_2_ plants were harvested, immersed in liquid nitrogen and stored at −70°C before DNA extraction.

### Phenotypic Evaluation

Five plants from each F_3_ family were selected randomly and their seed was used to represent the phenotype of individual F_2_ plants. As seeds matured, three capsules from the middle of the main capsule stem per plant were collected for phenotypic evaluation of each of the six generations. Seeds from each generation in each of the three replications were photographed using a digital Nikon camera in a darkroom. The RGB (red, green and blue) values of each picture were recorded for each of the three replications using the color capture tool in Adobe Photoshop. The average RGB value of each sample was used for statistical analysis.

### Segregation Analysis

Genetic analysis of the six populations was performed according to mixed major gene plus poly-gene genetic models [19]–[23]. The 24 genetic models could be divided into five model groups, i.e., inheritance controlled by one pair of major genes (A), two pairs of major genes (B), polygenes (C), one pair of major genes plus polygenes (D) and two pairs of major genes plus polygenes (E) (Table S1). The distribution parameters for seed coat color in each population were estimated using the iterated expectation and conditional maximization (IECM) method. The best-fitting genetic model was determined according to Akaike’s information criterion (AIC), a likelihood-ratio test and goodness-of-fit test [19]–[21]. The genetic effect of major genes and polygenes was estimated using the least squares method [21]–[22] based on the distribution parameters of each component in the optimal model.

### DNA Isolation and DNA Marker Analysis

Total genomic DNA of the parents and 260 F_2_ lines was extracted from 300 mg the young leaf tissue using the CTAB method [24]. 32 amplified fragment length polymorphism (AFLP) and 298 simple-sequence repeat (SSR) primers used in previous studies [17]–[18] were combined to generate thousands of AFLP and random selective amplification of microsatellite polymorphic loci (RSAMPL) primer pairs for screening polymorphic markers [17] (Table S2). DNA amplification and electrophoresis were performed according to Wei et al. [17].

### Linkage Map Construction

A total of 724 polymorphic primer pairs, including 49 SSR, 52 AFLP, and 623 RSAMPL primer pairs, were used for linkage mapping. A high-density linkage map was constructed with a total of 653 polymorphic loci using JoinMap ver. 3.0 [25]. Chi-square test was used to determine whether or not genotypic frequencies at each locus deviated from the expected segregation ratios of 1∶2∶1 (or 3∶1). All linkage groups (LG) were determined with an LOD score cut-off of ≥6.0. The expected length of the genome was estimated using the methods described by Fishman et al. [26] and Postlethwait et al. [27].

### QTL Detection

QTLs regulating seed coat color were analyzed using phenotypic data from the F_2_ and F_3_ generations respectively. Composite interval mapping (CIM) [28] and mixed linear composite interval mapping (MCIM) [29] were both used for QTL detection. In CIM, WinQTLCart 2.5 software (http://statgen.ncsu.edu/qtlcart/WQTLCart.htm) was run using Model 6 with four parameters for forward and backward stepwise regression, 10 cM window size, 5 control markers and a 1 cM step size [30]. The threshold was determined by permutations (1000 times). In MCIM, QTLnetworks 2.0 software (http://www.webtopicture.com/qtl/qtl-network.html) was run with genome scan parameters of a 10 cM testing window, 1 cM walk speed and 10 cM filtration window. Whether or not two adjacent test peaks represented independent QTLs was determined in this process. Critical F-values were calculated using the Permutation test. QTL effects were estimated using Monte Carlo Markov Chain (MCMC) [31].

## Results

### Seed Coat Color Phenotype

Field investigation showed that the phenotype of COI1134 (P_1_) was white seeded, while RXBS (P_2_), the F_1_ hybrid and the BC_2_ population were generally black. Seed color in the BC_1_ and F_2_ populations varied from black, intermediate to white (Figure S1). The RGB values of the six populations were consistent with their phenotypes (Table 1), and those of the P_1_ and P_2_ populations ranged between 120–150 and 20–50 respectively. The ranges of RGB values for the F_1_ and BC_2_ populations were similar to that of P_2_. The RGB values of the BC_1_ and F_2_ populations varied continuously from 20–150 and 20–140, respectively. The distribution of seed color in both backcross families (BC_1_ and BC_2_) shifted towards the recurrent parents.

**Table 1:** Distribution of RGB values for seed coat color in six genetic populations of sesame.

Test Replication	Population	RGB value														Plant number	Mean ± SD
		20∼	30∼	40∼	50∼	60∼	70∼	80∼	90∼	100∼	110∼	120∼	130∼	140∼	150∼		
I	P_1_											1	14	9	1	25	138.43±6.72
	P_2_	3	16	7	2											28	38.26±7.79
	F_1_	4	8	12	2	1										27	40.42±8.76
	BC_1_	2	12	52	12	16	6	14	21	17	24	19	10	8		213	82.57±34.22
	BC_2_	16	55	91	20	7										189	42.31±8.7
	F_2_	12	65	78	29	26	25	17	19	8	20	5	2	1		307	61.07±27.28
II	P_1_											3	13	9	3	28	139.08±7.8
	P_2_	3	17	8	2											30	38.25±7.26
	F_1_	3	9	13	4	1										30	42.55±8.62
	BC_1_	2	10	36	7	14	9	10	14	21	22	22	17	7	1	192	89.05±35.17
	BC_2_	13	57	90	18	7	1									186	42.41±8.98
	F_2_	12	69	69	28	26	19	17	19	7	14	7	1	1		289	59.84±26.86
III	P_1_											3	17	8	2	30	137.64±8
	P_2_	2	14	7	2											25	39.73±7.91
	F_1_	3	9	13	3	1										29	41.59±8.8
	BC_1_	1	11	37	7	7	6	9	18	21	20	10	5	3		155	82.32±32.57
	BC_2_	12	53	91	16	8										180	41.87±9.08
	F_2_	14	64	90	29	14	28	22	19	13	16	8	5	1		323	62.21±28.28

### Genetic Model Analysis

To determine a genetic model for the seed color trait, segregation analysis was performed in the six populations (with three replications) using a mixed genetic model (major genes+polygenes) (Table 2). The B-1, B-2, E-0, E-1 and E-2 models with smaller AIC values were selected as candidate models for further analysis. Fitness tests, including U_1_^2^, U_2_^2^, U_3_^2^, and Simirnov and Kolmogorov tests, were carried out. Results indicated that the number of significant parameters in the five models varied from 0 to 13 (Table S3). The E-0 model, with the least number of significant parameters (0), was selected as the optimal genetic model for seed color trait analysis. In this model, seed coat color is controlled by two major genes and polygenes with additive-dominant-epistatic effects (Table S1).

**Table 2:** AIC and maximum log likelihood values of 24 genetic models estimated using an iterated ECM (IECM) algorithm.

Geneticmodel	Replication					
	I		II		III	
	AIC	Max-likelihood	AIC	Max-likelihood	AIC	Max-likelihood
A_1	7000.79	−3496.40	6697.66	−3344.83	6566.77	−3279.39
A_2	7437.55	−3715.77	7114.38	−3554.19	6971.62	−3482.81
A_3	7633.27	−3813.63	7352.97	−3673.48	7173.73	−3583.86
A_4	7006.78	−3500.39	6707.43	−3350.72	6568.99	−3281.50
B_1*	6724.46*	−3352.23	6419.53*	−3199.76	6263.03*	−3121.51
B_2*	6716.10*	−3352.05	6418.85*	−3203.43	6266.58*	−3127.29
B_3	7481.31	−3736.66	7154.83	−3573.41	7007.95	−3499.98
B_4	7379.80	−3686.90	7035.43	−3514.71	6902.21	−3448.10
B_5	7602.86	−3797.43	7316.38	−3654.19	7145.88	−3568.94
B_6	7600.86	−3797.43	7314.38	−3654.19	7143.88	−3568.94
C_0	6935.07	−3457.54	6606.17	−3293.09	6507.41	−3243.71
C_1	6951.20	−3468.60	6619.09	−3302.54	6513.09	−3249.55
D_0	6764.56	−3370.28	6455.84	−3215.92	6334.18	−3155.09
D_1	6775.30	−3378.65	6455.27	−3218.64	6367.82	−3174.91
D_2	6773.30	−3378.65	6453.27	−3218.64	6365.82	−3174.91
D_3	6938.31	−3461.16	6602.60	−3218.64	6510.20	−3247.10
D_4	6773.29	−3378.65	6453.27	−3218.63	6365.82	−3174.91
E_0*	6713.55*	−3338.77	6404.31*	−3184.16	6269.96*	−3116.98
E_1*	6719.10*	−3344.55	6409.30*	−3189.65	6278.76*	−3124.38
E_2*	6712.40*	−3345.20	6413.95*	−3195.97	6266.03*	−3122.02
E_3	6952.98	−3467.49	6622.89	−3302.45	6513.73	−3247.86
E_4	6953.02	−3468.51	6620.88	−3302.44	6514.96	−3249.48
E_5	6955.12	−3468.56	6622.99	−3302.49	6517.00	−3249.50
E_6	6953.08	−3468.54	6621.52	−3302.76	6515.05	−3249.53

* These models were selected as candidate models with smaller AIC values.

### Genetic Effect Analysis

To investigate the genetic effects of two major genes, the first order genetic parameters were estimated using the least squares method (Table 3). Additive effects of the major genes (*a*, *b*) controlling seed color were 20.30 (d_a_) and 25.09 (d_b_) respectively, while individual values of the dominant effects were −35.94 (h_a_) and −9.35 (h_b_), showing a downward trend. The additive effect of gene *a* was lower than that of gene *b* (|da|<|db|), and the dominant effect of gene *a* was higher than its own additive effect (|ha/da| >1). In contrast, the dominant effect of gene *b* was lower than that of *a* (|ha|>|hb|) and its own additive effect (|hb/db| <1). The additive by additive (i) and additive by dominant (j_ab_) effects were low with average values of 2.97 and 7.76 respectively. The dominant by dominant (l) and dominant by additive (j_ba_) effects were high with average values of 17.87 and −18.72 respectively, while the dominant by additive (j_ba_) effect on seed color was decreased (−18.72).

**Table 3:** Estimation of 1^st^ order genetic parameters for seed color trait.

1^st^ orderparameter	Replication			Average
	I	II	III	
d_a_	20.13	20.16	20.60	20.30
d_b_	25.04	24.80	25.42	25.09
h_a_	−34.66	−38.00	−35.17	−35.94
h_b_	−8.62	−9.77	−9.65	−9.35
i	3.61	3.51	1.79	2.97
j_ab_	7.58	6.93	8.78	7.76
j_ba_	−18.45	−20.98	−16.74	−18.72
l	15.96	22.80	14.86	17.87
h_a_/d_a_	−1.72	−1.88	−1.70	−1.77
h_b_/d_b_	−0.34	−0.39	−0.37	−0.37

Estimation of second order genetic parameters are shown in Table 4. Average heritability values for the major genes in the BC_1_, BC_2_, and F_2_ populations were 89.30%, 24.00%, and 91.11%, respectively. Effects of polygenes were minor in the BC_1_ (5.43%) and F_2_ (0.89%) populations, and absent in the BC_2_ population.

**Table 4:** Estimation of 2^nd^ order genetic parameters for seed color trait.

2^nd^ order parameter	BC_1_				BC_2_				F_2_			
	Replication			Average	Replication			Average	Replication			Average
	I	II	III		I	II	III		I	II	III	
σ^2^_p_	1170.90	1236.68	1060.72	1156.10	75.74	80.59	82.49	79.61	744.35	721.61	799.51	755.16
σ^2^_mg_	1040.27	1108.23	948.62	1032.37	16.89	22.97	17.48	19.11	668.52	663.99	731.47	687.99
σ^2^_pg_	71.78	70.83	47.09	63.23	0.00	0.00	0.00	0.00	16.98	0.00	3.02	6.67
σ^2^	58.85	57.62	65.01	60.49	58.85	57.62	65.01	60.49	58.85	57.62	65.01	60.49
h^2^_mg_ (%)	88.84	89.61	89.43	89.30	22.30	28.50	21.19	24.00	89.81	92.02	91.49	91.11
h^2^_pg_ (%)	6.13	5.73	4.44	5.43	0.00	0.00	0.00	0.00	2.28	0.00	0.38	0.89

σ^2^_p_: Phenotypic variance.

σ^2^_mg_: Major gene variance.

σ^2^_pg_: Polygene variance.

σ^2^: Environmental variance.

h^2^_mg_ (%): Heritability of major gene(s).

h^2^_pg_ (%): Heritability of polygenes.

### Linkage Map Construction

**Figure 2:**
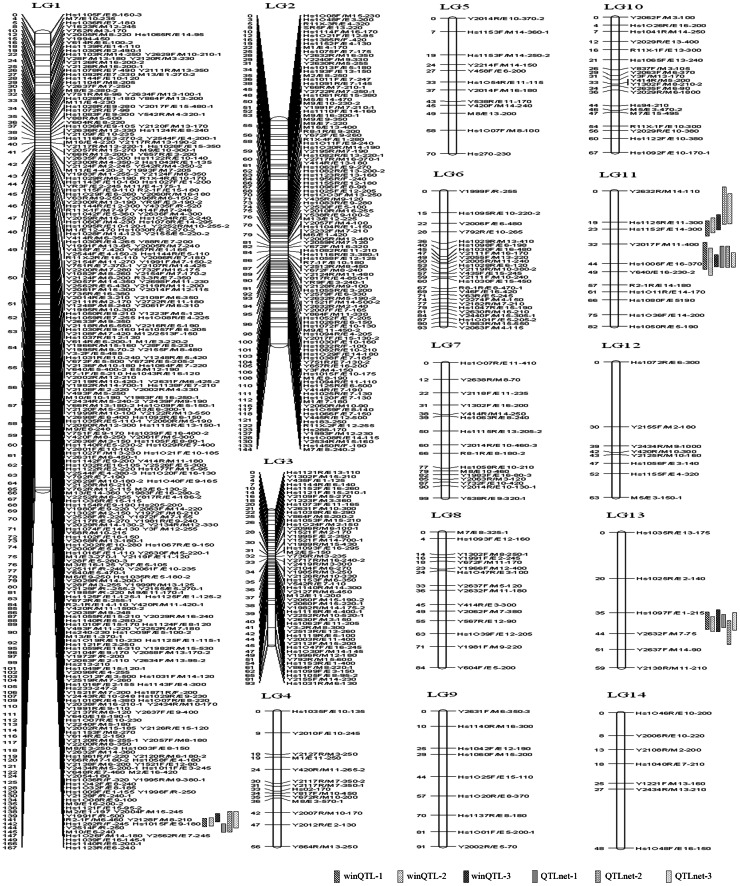
Distribution of QTLs for sesame seed color trait on our seame high-density genetic linkage map. 653 marker loci were distributed across the 14 linkage groups of our high-density genetic linkage map at an LOD threshold of 6.0. The cM distance of markers is shown on the left side of each LG. The name, amplicant length (bp) and band number of each marker are shown on the right side of each linkage group. An LOD peak value of >2.5 was considered to indicated a significant QTL interval. The four QTLs identified using two programs are designated as follows: winQTL-1, winQTL-2 and winQTL-3 represent the QTL loci from the F_2_ population and F_3_ families (Yuanyang) and _F3_ families (Pingyu), respectively using the winQTLCart program. QTLnet-1, QTLnet-2 and QTLnet-3 represent the QTL loci from the F_2_ and F_3_ family (Yuanyang) and F_3_ family (Pingyu), respectively, using networks program.

In order to construct a high-density linkage map, a subset of 724 polymorphic loci (49 EST-SSR, 52 AFLP, and 623 RSAMPL) were screened with an F_2_ mapping population of 260 plants at an LOD score of 6.0. The estimated size of the sesame genome is 1380.938 cM. A total of 653 marker loci (30 EST-SSR loci, 50 AFLP loci, and 573 RSAMPL loci) were assigned to 14 linkage groups. The linkage map covered 1,216.00 cM (88.06%) of the sesame genome (Figure 2). The average marker density in this map was approximately one marker per 1.86 cM, and the number of markers in each linkage group ranged from 6 (LG 14) to 345 (LG 1), with a mean of 46.64 markers per group. The length of linkage groups ranged from 47.54 cM (LG 14) to 166.57 cM (LG 1), with an average size of 86.86 cM. The groups with the highest and lowest average marker density were LG 1 (0.48 cM) and LG 13 (9.91 cM) respectively. In addition, segregation distortion (P<0.05) was observed for 79 (10.91%) markers in 12 linkage groups. LG 1 showed a high level of distortion, with 52 segregation distortion loci and a strong clustering tendency.

### QTLs for Seed Coat Color

Results for the QTLs analysis of the seed coat color trait using the CIM and MCIM methods are shown in Tables 5 and 6. Both methods indicated that the four QTLs, QTL 1-1, QTL 11-1, QTL 11-2 and QTL 13-1, were located in LG 1, LG11 and LG13. Using the CIM method, these QTLs were distributed in the regions between markers Y1991F/R and Hs1015F/E9, Hs1125R/E11 and Y2017F/M11, Y2017F/M11 and Y640/E16, and Hs1097F/E1 and Y2632F/M7, respectively (Figure 2). Among the three populations of the F_2_ and two F_3_ families (at Yuangyang and Pingyu), four QTLs explained 23.32–39.95% (QTL 1-1), 9.72–20.61% (QTL 11-1), 9.6–31.86% (QTL 11-2), and 12.8–30.56% (QTL 13-1) of the phenotypic variation, respectively (Table 5). QTL 1-1 made the highest contribution, followed by QTL 13-1.

**Table 5:** QTL analysis of seed color trait in F_2_ and F_3_ families using winQTLCart program.

Family	Linkagegroup	QTL name	Position (cM)	Marker interval (cM)	LOD	r^2^ (%)	Additive effect	Dominant effect
F_2_	1	1–1	141.8	140.9–141.9	39.32	0.3995	21.3146	−20.5064
	11	11-1	25.4	21.1–30.8	10.07	0.1020	13.2342	−10.1866
	11	11-2	38.2	32.2–47.8	8.30	0.0960	12.4177	−11.4117
	13	13-1	38.6	36.0–42.3	28.04	0.3056	18.6158	−18.8627
F_3_(Yuanyang)	1	1-1	141.2	140.5–141.8	20.36	0.2332	13.8790	−9.1352
	11	11-1	23.4	20.0–28.3	16.69	0.2061	11.8210	−12.5171
	11	11-2	43.2	36.8–51.2	14.74	0.2402	13.4322	−12.8673
	13	13-1	40.6	36.5–43.7	14.76	0.1943	10.0826	−13.0255
F_3_(Pingyu)	1	1-1	140.6	139.6–140.7	26.11	0.2750	14.0623	−12.4889
	11	11-1	21.6	18.6–27.9	8.42	0.0972	11.2305	−8.1756
	11	11-2	43.2	38.2–43.4	20.50	0.3186	16.2349	−12.5716
	13	13-1	39.6	34.7–43.7	11.91	0.1280	8.9181	−9.9547

**Table 6:** QTL analysis of seed color trait in F_2_ and F_3_ families using QTLnetworks 2.0 program.

Familiy	Linkagegroup	QTL name	Position(cM)	Marker interval(cM)	V(G)/V(P)	Additive effect	P-Value	Dominanteffect	P-Value	Additive effect (h^2^)	Dominant effect (h^2^)
F_2_	1	1-1	141.8	141.2–142.3	0.6989	19.7911	0.000000	−18.6479	0.000000	0.3003	0.0718
	11	11-2	43.2	35.2–43.8		10.4978	0.000000	−13.0854	0.000000	0.0556	0.0355
	13	13-1	38.6	36.6–41.6		18.1685	0.000000	−18.5278	0.000000	0.2102	0.0451
F_3_Yuanyang	1	1-1	141.2	139.6–142.3	0.5933	13.5107	0.000000	−9.5556	0.000000	0.194	0.0475
	11	11-1	20.6	0.0–23.4		7.2039	0.000000	−4.2611	0.036690	0.1390	0.0426
	11	11-2	43.2	35.2–43.8		4.4052	0.000607	1.8569	0.402717	0.1192	0.0264
	13	13-1	41.6	38.6–47.7		10.1818	0.000000	−11.5127	0.000013	0.1279	0.0269
F_3_Pingyu	1	1-1	141.2	139.6–141.8	0.6248	13.9201	0.000000	−12.0896	0.000000	0.2105	0.0699
	11	11-1	19.6	8.0–23.4		7.0118	0.000000	−4.4798	0.022901	0.2122	0.0128
	11	11-2	43.2	37.2–43.8		9.3244	0.000000	−4.7431	0.026869	0.1900	0.0136
	13	13-1	39.6	35.6–42.6		8.6601	0.000000	−10.3753	0.000059	0.0913	0.0213

Analysis of data from Yuanyang and Pingyu using the MCIM method indicated that the same four QTLs were detected in the F_3_ populations at both locations (Table 6). QTL 11-2 was the only one of the four QTLs that was not found in the F_2_ populations. All QTLs in the F_2_ and F_3_ families were distributed in the regions between markers Y2128F/M8 and Hs1015F/E9, Hs1125R/E11 and Hs1152F/E14, Y2017F/M11 and Hs1006F/E16, and Hs1097F/E1 and Y2632F/M7, respectively (Figure 2). These either overlapped or were adjacent to the corresponding regions identified using the CIM method. The heritability for this trait ranged from 59.33% to 69.89%. QTL 1-1, QTL 11-1, QTL 11-2 and QTL 13-1 contributed additive heritability of 19.4–30.03%, 13.9–21.22%, 5.56–19%, 9.13–21.02%, respectively. The dominant heritability for all QTLs ranged from 1.28–7.18% (Table 6). Positions of all QTLs in the F_3_ populations in the two environments were compared using the QTLnetworks 2.0 program (Figure S2).

To further confirm these findings, we performed an ANOVA using GLM procedure with color values as the dependents and markers of class variables (Table 7). Results indicated that the Hs1125R/E11-300 and Y2017F/M11-400 markers were located at 18.6 and 32.2 cM on LG11, respectively, and showed high R-square values (from 0.126 to 0.198) in both environments; the Hs1152F/E14-300 marker located at 23.4 cM on LG11 and showed lower R-square values (0.077 and 0.033) in the two environments.

**Table 7:** ANOVA of seed color trait in the F_3_ families.

Marker name	Position (cM)	Pingyu environment		Yuanyang environment	
		*P* value^a^	r^2^	*P* value^a^	r^2^
Y2632R/M14-110	0.000	0.0009	0.045	0.0360	0.018
Hs1125R/E11-300	18.637	<0.0001	0.198	<0.0001	0.177
Hs1152F/E14-300	23.410	<0.0001	0.077	0.0045	0.033
Y2017F/M11-400	32.185	<0.0001	0.147	<0.0001	0.126
Hs1006F/E16-370	43.823	<0.0001	0.100	0.0006	0.049
Y640/E16-230-2	48.793	0.0001	0.060	0.0100	0.028
R2-1R/E14-180	56.785	0.0168	0.024	0.0212	0.022

Note: a indicates the significance of the model.

In addition, we also investigated environmental effects on the seed color genotypes using the F_3_ datasets from the two environments (i.e., Yuanyang and Pingyu) and the MCIM method. Results indicated that the genotype×environment effect was not significant for seed coat color (data not shown). In conclusion, we were able to assign four QTLs for seed coat color in Linkage groups, LG1, LG11 and LG13. Alleles at all QTLs in the black-seeded parent (RXBS, P_2_) increased the tendency toward darker seed coat color.

## Discussion

Seed coat color in sesame is an important agronomic trait as it is associated with biochemical functions involved in protein and oil metabolism, antioxidant activity, and disease resistance [2]–[6]. Recent reports suggest that the seed coat color trait is a more suitable trait for estimating sesame evolution than geographic origin [7], since the direction of evolution in sesame was from wild species to black cultivars and then white cultivars [7], [32]. While exploring the genetic basis and identifying QTLs for seed coat color, we constructed a new sesame genetic map with 653 loci using an intraspecific cross between white and black seeded accessions.

### Genetic Analysis

In order to improve the precision of genetic analyses for quantitative traits, the use of a segregating population with more than 100 individuals is suggested or even required [19], [22]. More than one generation with several replications is also encouraged [23]. We therefore performed the segregation analysis on seed coat color using a large experimental group (more than 150 individuals from each segregating population) with three replications. Six populations (P_1_, P_2_, F_1_, BC_1_, BC_2_ and F_2_) and F_3_ families grown in two environments were used in this study.

The genetic effects and heritability of the gene(s) revealed that seed color is a complex quantitative trait in sesame: it is regulated by two major genes and polygenes with additive-dominant-epistatic effects (E-0 model). Seed coat color in sesame is primarily controlled by hereditary factors. More than 90.0% of the phenotypic variation in the BC_1_ and F_2_ populations is controlled by two major genes and polygenes, with minimal influence from environmental factors (<10.0%). Major genes in the BC_2_ population controlled 24.0% of the phenotypic variation. The same phenomenon of hereditary variation in different generations and populations has also been documented in tomato, cucumber, maize and other crops [33]–[36]. Further functional genomic studies are required to clarify the molecular mechanism controlling seed coat color.

### Genetic Linkage Map

Using the same cross as was used for the construction of the first linkage map, we herein constructed a high-density linkage map in sesame using 724 PCR-based DNA markers. Compared with the first map (data shown in brackets below) [17], this new map presents optimal features, e.g., (1) a more stringent criteria of LOD ≥6.0 (LOD ≥4.0) was used for map construction; (2) larger F_2_ segregating populations of 260 (96) individuals were used; (3) the map is comparatively saturated with 653 (220) markers in 14 (30) linkage groups; (4) the average marker distance is 1.86 cM (4.93 cM); and (5) the estimated sesame genome is 1,380.94 cM (1,232.53 cM) and genome coverage is 88.06% (76.00%). We believe that more polymorphic genic SSR and SNP markers, and EST-SSR, AFLP and RSAMPL markers, will be validated and used for higher-density sesame linkage map construction within the near future [18] (Zhang H. et al., unpublished data).

In this study, 79 loci with distorted segregation were detected in 12 out of the 14 linkage groups and the degree of clustering of marker loci showed marked variation in the new sesame map. A similar deviation from Mendelian segregation ratios were also observed in our previous study [17]. Many factors, including technical artifacts in genotyping [37]–[41], chromosomal rearrangements [39]–[40] and markers from transposable elements [42], may have contributed to this effect. Varying degrees of clustering for AFLP markers have been reported in different crops including rice [15], barley [43], maize [44], ryegrass [45], tomato [46], potato [47], and *Eucalyptus globulus* and *E. tereticornis* [48]. Differences in the level or location of DNA polymorphisms, rates of recombination, copy number variation of specific genomic sequences or sampling errors are the main factors influencing marker distribution [47]–[48].

### QTLs and Genes for Seed Coat Color

In this study, we identified four stable QTLs for seed coat color in sesame and estimated their gene effects. In the F_2_ population, QTL 1-1 and QTL 13-1 played major roles, explaining 39.95% and 30.56% of the phenotypic variation (Table 5), and having additive effects (h^2^) of 30.03% and 21.02% (Table 6), respectively. QTL 11-1 and QTL 11-2 are regarded as polygenes due to their comparatively lower contributions. This result is consistent with the results from classical genetic analysis (Table S1). The seed color trait is relatively stable and is not affected by environmental factors [49]–[51]. In other crops, the number of genes controlling the seed coat color trait is variable; for example, the seed color trait is regulated by a single gene in flax [52], watermelon [53], and lettuce [54], while two independent loci were found in lentil [55] and biennial white sweet clover [56], and at least three genes are involved in controlling the trait in pea [57] and capsicum [58].

Using the winQTLCart program and F_3_ population data, QTL 11-1 and QTL 11-2 contributed 20.61% and 24.02%, respectively, of the phenotypic variation in Yuanyang (Table 5), making a similar contribution to that of QTL 1-1 and QTL 13-1. Furthermore, a similar situation was also observed in Pingyu when data was analyzed using the networks program (Table 5 and 6). We thus suggested that QTL 11-1 and QTL 11-2 may play major roles and have comparable effects to QTL 1-1 and QTL 13-1.

It is noteworthy that the QTL 11-1 and QTL 11-2 in LG 11 are quite close to each other. Whether these QTLs are independent or two parts of one larger QTL is a question worth consideration. He et al. (2001) reported that independence between two QTLs is based on heritability, marker density and sample size [59]. In F_2_ or F_3_ populations, if the heritability of a QTL is 10%, the marker density is 15 cM and the sample size is 300, the likelihood of detecting two adjacent QTLs would be 80% [59]. In our study, results showed that the two QTLs in LG11 had a heritability greater than 10% (Tables 5, 6), a QTL distance of greater than 20 cM (Figure S2) and a marker density in LG11 of greater than 15 cM. We therefore concluded that there are two QTLs in the LG11 region. To further confirm this hypothesis, we performed an ANOVA using the GLM procedure with color values as the dependents and markers of class variables (Table 7). Results obtained also supported the existence of two QTLs in the LG11 region.

Several genes related with seed coat color have been cloned from *A. thaliana*, *Brassica* and *Glycine max* using fine-mapping, T-DNA insert mutation and homology-based cloning strategies [60]–[62]. Due to the association between seed coat color and important biochemical functions [2]–[7], [63], we will continue to perform gene cloning and functional research on seed coat color traits in our ongoing Sesame Genome Project (www.sesamum.org) [64].

### Conclusion

We have assembled a high-density linkage map of sesame with 653 marker loci in 14 LGs. We have shown that seed coat color is controlled by two major genes with additive-dominant-epistatic effects plus polygenes with additive-dominant-epistatic effects, and detected four QTLs, QTL1-1, 11-1, 11-2 and 11-3 which are distributed in three linkage groups. Our results for segregation analyses and QTL detection for sesame seed coat color were consistent. Location of genes controlling seed color on the linkage map should be useful for gene isolation and functional genomics research. This first QTL mapping study in sesame provides a foundation for further genetics and molecular marker-assisted selection (MAS) breeding research.

## Supporting Information

Figure S1 **Seed coat color variation in six populations.** In this figure, seed color images (a-n) represent the corresponding RGB values for the14 grades (20–150) (Table 1). A series of seed coat colors in sesame populations are included. (a) represents the black-seeded parent and (n) the white-seeded parent.
(TIF)

Figure S2 **QTLs detected in the F_3_ populations in two environments using QTLnetworks program 2.0.** A: QTL estimation in F_3_ populations from Pingyu. B: QTL estimation in F_3_ populations from Yuanyang.
(TIF)

Table S1 **Genetic models for P_1_, P_2_, F_1_, BC_1_, BC_2_ and F_2_ population analysis.** The genetic models are cited from Gai et al. [20] and Zhang et al. [23] and were divided into five model groups, i.e., inheritance controlled by one major gene, two major genes, polygenes, one major gene plus polygenes and two major genes plus polygenes.
(DOC)

Table S2 **AFLP and SSR primers used for linkage map construction.** 32 AFLP and 298 SSR primer pair combinations were used to screen for polymorphic primer pairs in genetic linkage map construction. The 50 AFLP primer pairs anchored onto the map were screened from combinations of the 32 AFLP primers, while the 30 SSR primer pairs anchored onto the map were obtained from previous research [17]–[18], and the 573 RSAMPL primer pairs anchored onto the map were screened from combinations of the 32 AFLP and 298 SSR primers.
(DOC)

Table S3 **Fitness tests of five candidate genetic models for seed coat color analysis.** The number of significant parameters, correlated with the adaptation level of the models, varied from 0–13. The E-0 model with the least number of significant parameters (0) in the three replications was selected from the five candidate models as the optimal model and was used for seed coat color analysis. *indicated significance at p = 0.05.
(DOC)
