# Supplementary figures and images for: Genetic Analysis and QTL Mapping of Seed Coat Color in Sesame (Sesamum indicum L.)
Source: PLoS One. 2013 May 21;8(5):e63898. doi: 10.1371/journal.pone.0063898 (PMC3660586; doi:10.1371/journal.pone.0063898)

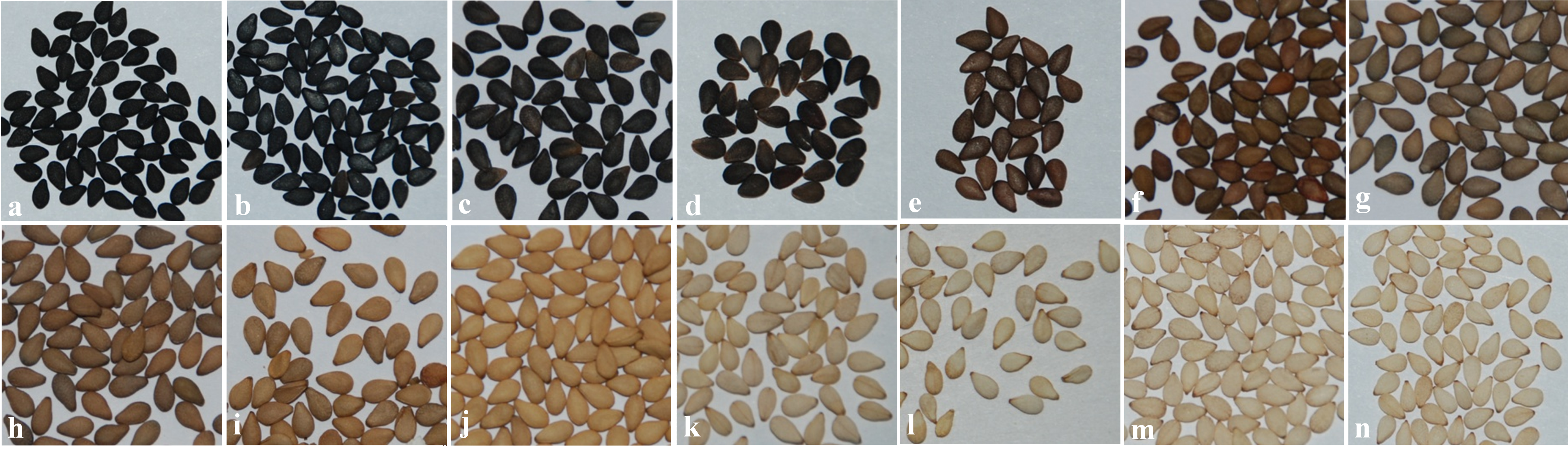

Supplement: Figure S1 — Seed coat color variation in six populations. In this figure, seed color images (a-n) represent the corresponding RGB values for the14 grades (20–150) (Table 1). A series of seed coat colors in sesame populations are included. (a) represents the black-seeded parent and (n) the white-seeded parent. (TIF) [file pone.0063898.s001.tif]

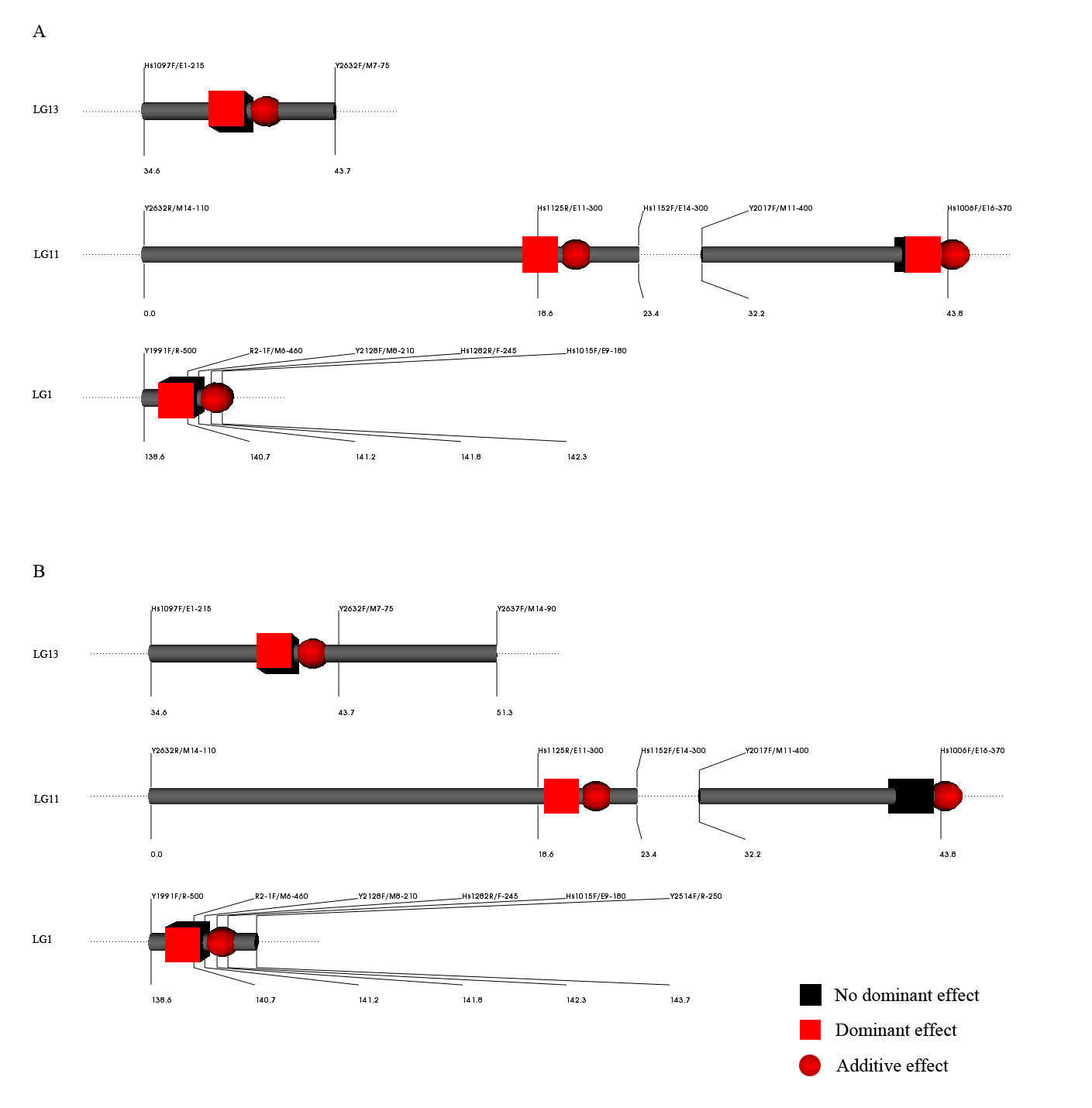

Supplement: Figure S2 — QTLs detected in the F3 populations in two environments using QTLnetworks program 2.0. A: QTL estimation in F3 populations from Pingyu. B: QTL estimation in F3 populations from Yuanyang. (TIF) [file pone.0063898.s002.tif]
