# Supplementary material for: Genetic Analysis and QTL Mapping of Seed Coat Color in Sesame (Sesamum indicum L.)
Source: PLoS One. 2013 May 21;8(5):e63898. doi: 10.1371/journal.pone.0063898 (PMC3660586; doi:10.1371/journal.pone.0063898)
Supplement: Table S1 — Genetic models for P1, P2, F1, BC1, BC2 and F2 population analysis. The genetic models are cited from Gai et al. [20] and Zhang et al. [23] and were divided into five model groups, i.e., inheritance controlled by one major gene, two major genes, polygenes, one major gene plus polygenes and two major genes plus polygenes. (DOC) [file pone.0063898.s003.doc]

**Table S1** **Genetic models for P_1_, P_2_, F_1_, BC_1_, BC_2_ and F_2_ populations analysis.**

| **Code** | **Model type** | **Number of the**  **major gene locus** | **No. of component distributions and implication of major gene** | **Implication of polygenes** |
| --- | --- | --- | --- | --- |
| 1 | A-1 | 1 | 10, additive-dominant major gene | No polygenes |
| 2 | A-2 | 1 | 10, additive major gene | No polygenes |
| 3 | A-3 | 1 | 8, positive complete dominant major gene | No polygenes |
| 4 | A-4 | 1 | 8, negative complete dominant major gene | No polygenes |
| 5 | B-1 | 2 | 20, additive-dominant-epistatic major gene | No polygenes |
| 6 | B-2 | 2 | 20, additive-dominant major gene | No polygenes |
| 7 | B-3 | 2 | 20, additive major gene | No polygenes |
| 8 | B-4 | 2 | 14, equal additive major gene | No polygenes |
| 9 | B-5 | 2 | 12, complete dominant major gene | No polygenes |
| 10 | B-6 | 2 | 10, equal dominant major gene | No polygenes |
| 11 | C-0 | 0 | 6, no major gene | Additive-dominant-epistatic polygenes |
| 12 | C-1 | 0 | 6, no major gene | Additive-dominant polygenes |
| 13 | D-0 | 1 | 10, additive-dominant major gene | Additive-dominant-epistatic polygenes |
| 14 | D-1 | 1 | 10, additive-dominant major gene | Additive-dominant polygeness |
| 15 | D-2 | 1 | 10, additive major gene | Additive-dominant polygenes |
| 16 | D-3 | 1 | 8, complete dominant major gene | Additive-dominant polygenes |
| 17 | D-4 | 1 | 8, negative complete dominant major gene | Additive-dominant polygenes |
| 18 | E-0 | 2 | 20, additive-dominant-epistatic major gene | Additive-dominant-epistatic polygenes |
| 19 | E-1 | 2 | 20, additive-dominant-epistatic major gene | Additive-dominant polygenes |
| 20 | E-2 | 2 | 20, additive-dominant major gene | Additive-dominant polygenes |
| 21 | E-3 | 2 | 20, additive major gene | Additive-dominant polygenes |
| 22 | E-4 | 2 | 14, equal additive major gene | Additive-dominant polygenes |
| 23 | E-5 | 2 | 12, complete dominant major gene | Aadditive-dominant polygenes |
| 24 | E-6 | 2 | 10, equal dominant major gene | Additive-dominant polygenes |

The genetic models are cited from Gai et al. [20] and Zhang et al. [32] and were divided into five model groups, i.e., inheritance controlled by one major gene, two major genes, polygenes, one major gene plus polygenes and two major genes plus polygenes.
