# Supplementary material for: Genetic Analysis and QTL Mapping of Seed Coat Color in Sesame (Sesamum indicum L.)
Source: PLoS One. 2013 May 21;8(5):e63898. doi: 10.1371/journal.pone.0063898 (PMC3660586; doi:10.1371/journal.pone.0063898)
Supplement: Table S2 — AFLP and SSR primers used for linkage map construction. 32 AFLP and 298 SSR primer pair combinations were used to screen for polymorphic primer pairs in genetic linkage map construction. The 50 AFLP primer pairs anchored onto the map were screened from combinations of the 32 AFLP primers, while the 30 SSR primer pairs anchored onto the map were obtained from previous research [17]–[18], and the 573 RSAMPL primer pairs anchored onto the map were screened from combinations of the 32 AFLP and 298 SSR primers. (DOC) [file pone.0063898.s004.doc]

**Table S2. AFLP and SSR primers used for linkage map construction.**

| **Primer code** | **Primer sequence （5' end to 3' end）** |
| --- | --- |
| E1 | 5’ GACTGCGTACCAATTCGAA3’ |
| E2 | 5’ GACTGCGTACCAATTCGAC3’ |
| E3 | 5’ GACTGCGTACCAATTCGAT3’ |
| E4 | 5’ GACTGCGTACCAATTCGAG3’ |
| E5 | 5’ GACTGCGTACCAATTCGGG3’ |
| E6 | 5’ GACTGCGTACCAATTCGGA3’ |
| E7 | 5’ GACTGCGTACCAATTCGGC3’ |
| E8 | 5’ GACTGCGTACCAATTCGGT3’ |
| E9 | 5’ GACTGCGTACCAATTCGCC3’ |
| E10 | 5’ GACTGCGTACCAATTCGCT3’ |
| E11 | 5’ GACTGCGTACCAATTCGCA3’ |
| E12 | 5’ GACTGCGTACCAATTCGCG3’ |
| E13 | 5’ GACTGCGTACCAATTCGTT3’ |
| E14 | 5’ GACTGCGTACCAATTCGTA3’ |
| E15 | 5’ GACTGCGTACCAATTCGTG3’ |
| E16 | 5’ GACTGCGTACCAATTCGTC3 |
| M1 | 5’GATGAGTCCTGAGTAACAA3’ |
| M2 | 5’GATGAGTCCTGAGTAACAT3’ |
| M3 | 5’GATGAGTCCTGAGTAACAC3’ |
| M4 | 5’GATGAGTCCTGAGTAACAG3’ |
| M5 | 5’GATGAGTCCTGAGTAACCA3’ |
| M6 | 5’GATGAGTCCTGAGTAACCG3’ |
| M7 | 5’GATGAGTCCTGAGTAACCT3’ |
| M8 | 5’GATGAGTCCTGAGTAACCC3’ |
| M9 | 5’GATGAGTCCTGAGTAACTT3’ |
| M10 | 5’GATGAGTCCTGAGTAACTA3’ |
| M11 | 5’GATGAGTCCTGAGTAACTG3’ |
| M12 | 5’GATGAGTCCTGAGTAACTC3’ |
| M13 | 5’GATGAGTCCTGAGTAACGA3’ |
| M14 | 5’GATGAGTCCTGAGTAACGC3’ |
| M15 | 5’GATGAGTCCTGAGTAACGG3’ |
| M16 | 5’GATGAGTCCTGAGTAACGT3’ |
| Hs02F | CCATTAAATTCTTGCTCCCC |
| Hs02R | CTGGTCGTATGCAGCATCTT |
| Hs21F | CGGAATTCCTGAAAGAAGGA |
| Hs21R | CAGTGAATTTCTCAACCCGA |
| Hs53F | GAAGCTTGAAGAGAGGAGGG |
| Hs53R | ATGGAACTTCTCCGATCACC |
| Hs94F | CATGTGTTCTCTCCCACCAC |
| Hs94R | TCTTGACCATGTTTTCCACC |
| Hs183F | TTGAAGGCGATGTAGGTCAG |
| Hs183R | CTGGTGATTCAGCCATTTTG |
| Hs189F | CTCCAACCCCCATAAATCAC |
| Hs189R | GCTTCTGGAGAGGAGATTGC |
| Hs205F | GATGTGATGGTGGTGAGAGC |
| Hs205R | GCTATGCGTTGAATGAAGAC |
| Hs207F | TCCTCACCATCATTGCTCAT |
| Hs207R | CTAAACTCAAGAAAGCGGGG |
| Hs213F | CCTCTCCTCTTCCGATCTTG |
| Hs213R | TCTGTCGTGGAAGAATGAGC |
| Hs214F | GGGAAACCCAACTCAAAAGA |
| Hs214R | TGGAAGCAGACAAGTAAGTGTG |
| Hs216F | TGAGAGAGGTTAATTGGGGG |
| Hs216R | TGGCTCCCATGTATTTACCA |
| Hs226F | AGGGGAGAATTCAGGTGTTG |
| Hs226R | GGGCACTGGAGATTTCTGTT |
| Hs233F | CGTCCCGTGTTGTCTCTATG |
| Hs233R | GCGGAGAATATGCCGTTATT |
| Hs235F | TTGCAGTAAAGCAATGGAGG |
| Hs235R | ATGCAGCATCAACTCCTCAG |
| Hs240F | TGGGCTTGGATTACTGATGA |
| Hs240R | ATGAGAAGGTGGTGCAGTGA |
| Hs259F | AAAGCCTCCCATACGATCAC |
| Hs259R | ACCGACGGAAACAACTAAGC |
| Hs270F | TTCGTGAAAAGGAGTTGTGC |
| Hs270R | CAGAGGTCACCATTGACGAG |
| Hs288F | CGACTTCGATGAATGTGAGC |
| Hs288R | CGAGGAAGGAGGTGACTAGC |
| Hs1001F | GAGGGCATCAACAACATCAC |
| Hs1002R | TAGCAAGAGCTCCAACGAGA |
| Hs1003F | AAATCATCCAAGACCCAAGC |
| Hs1006F | CGCGAATCAAAGTTCACACA |
| Hs1007F | TCCATCACCTCCTCATCAAA |
| Hs1007R | CCGAGGACACAAGGAATCTT |
| Hs1008F | TCTTCTCGTCCTTGCACAAC |
| Hs1008R | GGCTTCCTCCATTTGTTCAT |
| Hs1009F | ATGCTCTATTCCTTGCACCC |
| Hs1010F | CATAAAGGGCTACGGGGATA |
| Hs1010R | GGCTACAAAGAGGCATCTCC |
| Hs1011F | GGATAGGGTGAGGGCTGATA |
| Hs1011R | CGTGGCAATAGACCAAATCA |
| Hs1012F | TAGCCGTATTTGCGAGTCTG |
| Hs1012R | ATGGTAGAGTGCACGCAGAG |
| Hs1013F | GTGGTGAAGAAGGCACATTG |
| Hs1014F | CTCGGAATTGGATGGAAGTT |
| Hs1015F | GGTGTCGTTGATGTTTGAGG |
| Hs1016F | CTGCAAACAGGAAGGTTTCA |
| Hs1019R | CAGGCTTTACACTCCAGCAA |
| Hs1020F | TGGTGGCTCAAAACAAGTTC |
| Hs1020R | ACTCTCGACGGAGAAGGAGA |
| Hs1021F | AGATTTGGCAGCTGGTTCTT |
| Hs1024F | GGAAGCGAGAATCAATAGGC |
| Hs1025F | AGACTAGCAACAGAGGGCGT |
| Hs1025R | CTGGTTGAATTTGCTGCTGT |
| Hs1026F | TGCAGTCTTTGCTTTTGTCC |
| Hs1026R | TCCATCTCTCAATCCACAGC |
| Hs1027F | ATGGAGAGGCTCTATGTGGG |
| Hs1027R | TTGAGAAGTTGCGATGAAGG |
| Hs1028F | TACCTGTGCCTTTGAAGCAG |
| Hs1028R | CATCAGTGGTGGATCTGAGG |
| Hs1029F | CTTTCCAGAGAGAGAAACCCA |
| Hs1029R | ACCCTGGCGTAAAAGACTTG |
| Hs1030F | ACTGCCCCAAGACCACTAAG |
| Hs1030R | ATTTGGGAGCATAGGGAATG |
| Hs1031F | GCAGCCAGAATTTCAAGTCA |
| Hs1031R | CCATGAAGGAACTGAAGGGT |
| Hs1032F | CTGAGGCACCAAAGACAAGA |
| Hs1032R | TCCAACTCCGGGTTTATGAT |
| Hs1034R | CTCGAATGATGGTGGACTTG |
| Hs1035F | GTTCCCCTACTTCTCCACCA |
| Hs1035R | TACTTGATCCTTTGCCCCTT |
| Hs1036F | GCCTGCTTAGCTGCCTTTAG |
| Hs1036R | CAGACGGAGATGCAGATTGT |
| Hs1037R | AGTTTTGATGCTCCCCAAAG |
| Hs1038F | GAGGCTGCAGGCATACATAA |
| Hs1038R | GCATGTCAGCAGGAGGATAA |
| Hs1039F | GGCCGAACTTGATCAGAAAT |
| Hs1040F | GCAGAGGGATCGGAGTTATG |
| Hs1040R | TGGGTATCCTTGTGCAGTGT |
| Hs1041R | CCACCACCTCCACCTCTATT |
| Hs1042F | TAGTCGGGTTGTTGTTGAGC |
| Hs1043R | TATCCAACATTAGCAGGGGG |
| Hs1046R | GCTGACAGAACTGCTAACGG |
| Hs1047F | GGTGGGTCCTTGTGCTATTT |
| Hs1047R | AACCTGGAGAACCCAATGAG |
| Hs1048F | TCTACAAAACCTGCCCCTTT |
| Hs1049F | GGTGGGTATGTGTTCCATGA |
| Hs1050R | CTTTCCCCCGGCTTTTTAT |
| Hs1052F | GGTGCGTTGAGTGGTTCAT |
| Hs1053F | GGGAGGGTTTACCTTTCTCC |
| Hs1054R | GCTGATCGTTGATCATGTCC |
| Hs1055R | ATTGTGGATCCCTTGAGCAT |
| Hs1056F | TCAATCAAGCTAACCCACCA |
| Hs1057F | CGGGCATCATTTTTCTCTG |
| Hs1058F | CTTTCTTCCCTCCCCTCTTC |
| Hs1058R | TCATTTGCGAGGAGTTTGAG |
| Hs1059F | ATTAATGGCTCAACCCGAAG |
| Hs1059R | ATTCAGTACCAGTGGGAGGC |
| Hs1060F | ATTTCACCCCAGCTCAAGTC |
| Hs1061R | GCATGGAGAGTACGGGAGAT |
| Hs1062F | ATTCTTCCCGGTACAACGTC |
| Hs1063F | TACAATTCACTCCAGGACGG |
| Hs1063R | GGTTTGAGTTGCCTACGGAT |
| Hs1064F | GCAAACACCACCACAAAGTC |
| Hs1065F | GGCCAACTAATGCAGTCAAA |
| Hs1065R | CAGTTAGGCTGCTCGTTGAA |
| Hs1066F | ACTCTCCAAGCTTCTTCCCA |
| Hs1067R | ACAACTTCAAACTAGCCGGG |
| Hs1069R | TGGTAGACCAGGGTCCTCTC |
| Hs1070R | GAAGCATCTGCATCTCTCTCA |
| Hs1072F | ATTTTCTCCACTCCCCCTTT |
| Hs1072R | GAGAAGCTTCAACCACAGCA |
| Hs1073F | ACAGCTTCCACATCATCCAA |
| Hs1074F | GCCGAAGAGTATTACGGGAG |
| Hs1075F | GCCCTTCCTTCTTTCTCCTT |
| Hs1077F | CATCGGCATCCTCTTCTTCT |
| Hs1078R | CGTCAGCTTCTCTTGCTTTG |
| Hs1080F | GGCTAGGGGTGACTTCCATA |
| Hs1082R | ATGAGAAGCTTGGGTCCTGT |
| Hs1083F | GGGAAAGGAGAGAACGACAA |
| Hs1083R | GATTTCTGCTGTAATGGCCC |
| Hs1084F | AAATATTGAAGGCACGGAGG |
| Hs1087R | CTGATTTTGGTGGTGCTGAT |
| Hs1091R | TCCAACTGCCTACATGGAAA |
| Hs1092F | TTGGACCTTGAAGATGATCG |
| Hs1092R | GAGGAGGAATCCAGTGAGGA |
| Hs1093F | AGCTTTTGCTGCTGCCTT |
| Hs1094R | GTGCGAGAATCGGTTTTGTA |
| Hs1095R | GCATCTTTGTGCTCATTGCT |
| Hs1096F | ATTTTCAGGCCTTGACTCGT |
| Hs1097F | TTCATCATCGTCACCACCTT |
| Hs1098F | GAGATCGTGGAAGGTTTGGT |
| Hs1099F | TCTGAGGACGATGAATTGGA |
| Hs1102F | CCACTGCCTGTCTAGGGAAC |
| Hs1102R | CAAGATCCAGAACTCCAGCA |
| Hs1103R | CCAGGCGTCCTCATATACCT |
| Hs1104F | CCTACCACAACTGCACCATC |
| Hs1104R | ATGACTTGCTTGTGGCTGAG |
| Hs1105F | CCTTGCACCTCTAACCCTTC |
| Hs1110F | AAGCAAGAGAGCAGCAACAA |
| Hs1114F | CTAGCGTTGCCTCCATTGTA |
| Hs1115F | GGATTCTTCGTGGTACGCTT |
| Hs1115R | TCCAGGGAAGTAAGAGGAGG |
| Hs1116R | GGGGCTGGTAACTTGGACTA |
| Hs1119R | TTTCCCTCCCATCACTTCAT |
| Hs1120F | CAACACTCATTCATCCACCA |
| Hs1121F | TCAGAAGCAGAAGTTGTGACTG |
| Hs1121R | TCGTAGTTCAGGATTGACGC |
| Hs1122F | TGGAGGTCTGAACGGATACA |
| Hs1122R | ACAGGCGGTCTTCTCTCAGT |
| Hs1123R | GACCAAGAGGGGAAAGATGA |
| Hs1124F | GATGGCACCTGAACCTTCTT |
| Hs1124R | AGAGCCATTAATCCCATTCG |
| Hs1125F | TTCAGCCCACTTCACTTCAG |
| Hs1125R | ACGCTCGCACTTCTTTTTCT |
| Hs1136F | GAGCCCAATCCTTCAAATGT |
| Hs1137R | TTCCCCTGAAAATATCCTCG |
| Hs1138F | GCTCTTCTTCTCAGACCGCT |
| Hs1139R | CCTTCTCTCACTACGCTCCC |
| Hs1140F | GGAGCGTAGTGAGAGAAGGG |
| Hs1140R | GTGCCATGTCAAAACCACTC |
| Hs1142F | GCAAAAAGAGCAGAGAACCA |
| Hs1143F | AACCCTACGTGCACAAGTCA |
| Hs1144F | CCACCTCCCAAGAAACCTTA |
| Hs1144R | AAGCCCTCAGAATCGTCATC |
| Hs1152F | ACCATATCGGGTGCCATATT |
| Hs1153F | AAGGAGCTGAAGAAGCCAAA |
| Hs1153R | GTTGCATCATTTCATGGAGG |
| Hs1154R | GGTACAACGCAGAGAGCAAA |
| Hs1155F | AAGTCGATCTCCACGCTTTT |
| Hs1155R | GTGCACTGGAGTTGCTCAAT |
| Hs1163F | CATCAGGCCAATCCCTATCT |
| Hs1163R | ATGCTATCCCCACCACTCTC |
| Hs1282F | CGTAAACGTCGTCGTCTTGT |
| Hs1282R | CCCAGAAAATCCAGAGAAGG |
| Hs1385F | GAGAAAGTTCAGGTGTGCGA |
| Hs1385R | TTGTCCAGAAGCCCTTTCTT |
| Hs1450F | GAGTGAGCATTGGGATGATG |
| Hs1450R | GTACAAGATGAGCGGCAAAC |
| Hs1514F | CCATGAGTGTTGGCTTTCTC |
| Hs1514R | CTGCTGTAAATGACGGGATG |
| Hs1792F | ACCCTGGCGTAAAAGACTTG |
| Hs1792R | TCCAGAGAGAGAAACCCAAGA |
| Hs1832F | TAACGGGTAGGTTTCGATCC |
| Hs1832R | AGAAAGGCAAAGCAGAGGAG |
| Hs1871F | GCATTTCAGGTGGACAAATG |
| Hs1871R | TAATGAGCAGAGGCACAAGC |
| Hs1956F | CACAGTTACCATGGGCAAAG |
| Hs1956R | ACACCCATATTTCCAGGCAT |
| Hs1961F | GCAAGAAGTCAGCTGATGGA |
| Hs1961R | TTCACATTCAAGTCAAGCCC |
| Hs1972F | TTCGGTGGCATTAGCTGTAG |
| Hs1972R | TTGTGGGCATAGGTGGTAGA |
| Hs1977F | ACAGTATTTGGGACTTGCCC |
| Hs1977R | GCCGTTTCCTTGTCTGATTT |
| Y1966F | ACAGCACTTACCCCAAAGGA |
| Y1966R | TGGGAGCCAACTTTCATTCT |
| Y1968F | CAGAATTCATTCTTCAACAACTCTTC |
| Y1968R | CGTGTTCCATCCCGTAACTT |
| Y1972F | CACGGAAGCAGCTCATCAT |
| Y1972R | CCTGCCGACATGACTACAAC |
| Y1980F | TGAGAGGAATTGGATTGGAAA |
| Y1981F | TCCCTTATTTGCAAGCAACC |
| Y1981R | AGGACAAGATCCACGGTGAG |
| Y1982R | GCGTTTCCCATTCCTAAGAC |
| Y1983F | AATTGAGAAAAAGAAAATGTTTGAA |
| Y1983R | AACGTTGAAGGTCCAACCAG |
| Y1985F | TAGCTCTCGCCGTTCTGTTT |
| Y1985R | CTCCTCCTCGAACCTTCCTT |
| Y1991F | TCCCCCAAATTTCACAAAAA |
| Y1991R | AGAGTAGGTTGCGCTCCTCA |
| Y1993F | TCTCCGACCATCAAAACCAT |
| Y1995R | GAAGCAATGCCTCCTTCAAC |
| Y1996F | TAGGAAGAGGCATGTTCACG |
| Y1996R | CATCTCCACATCTTGCATCG |
| Y1999F | CATTAGGCCTTGTCCATGCT |
| Y2000F | CCATCTCATCTCATCTCCCTTC |
| Y2003R | GCTAGTGAGCGGTTTCTTGG |
| Y2004F | AACCCCACTAGGCGAAGAAT |
| Y2005R | GTGATGCAGCTGAAGTGGAA |
| Y2006F | CTTACACGCCATGGCTTCAC |
| Y2006R | TTCCCGAACTTCCTCTTGAA |
| Y2007F | GACGACGCTAAGTCCGAATC |
| Y2007R | AGGGGTTAAGTGAGGCTGGT |
| Y2010F | ATTTGGCTGCTGCTCTCTTC |
| Y2010R | ATGGAATGGTGGAGTGGTGT |
| Y2012R | ACTTGACAGCCATGGGAAAG |
| Y2014F | AAGGATTGGCAGGATCATTG |
| Y2014R | TCGCCACCTATCTGTCATTG |
| Y2017F | CAAACTTGAACCACGACAGC |
| Y2055F | TTCTGTGGCACTCGTAGTCG |
| Y2056F | ACGGAGCCTGTATCATCAGC |
| Y2056R | GTGGCATGCATAGTCAGTCG |
| Y2057F | CTTTGATGGTGGCAATTGTG |
| Y2057R | GGGGTGGGGACTTGTACTTA |
| Y2058F | TAGGCATTGCCAATTTGTGA |
| Y2058R | TTCTGTGGCACTCGTAGTCG |
| Y2059R | ATTCTTTGCGCCTCTTTGTG |
| Y2060F | AACCCCATTTCTTCGCTGTA |
| Y2060R | CACTCCCATCCCACCATACT |
| Y2061F | ACGGCTCCAAATGTGTTTGT |
| Y2062F | CGCCTCCTTTATCACCATGT |
| Y2062R | GGGTTTTGCGGTTAAGTTGA |
| Y2063F | ACAGGCAGCAACAAAAGTCC |
| Y2063R | AAGGCAAAGCCCGAAATAAC |
| Y2107R | TCGCCACCTATCTGTCATTG |
| Y2109F | ACGGCTCCAAATGTGTTTGT |
| Y2111F | TGGATGCACCACTAGGAACA |
| Y2111R | AATCCCTTTTCTCACTGCTCA |
| Y2117R | AGGAGCCTTTGGTTGAGGAT |
| Y2118R | AAACGATCGACGACTGTTCA |
| Y2119F | CGTGGTCGTGCTTCTTCTTC |
| Y2119R | ATCCCATTCCGAGCACAA |
| Y2120F | GTACTCTTCCTCCGCCTCCT |
| Y2120R | CGGTCACCTGAATTTCCATC |
| Y2122R | TTTCCTGCCAACTTTTCTGG |
| Y2124F | TCCTCAGAGCTGCACATTTTT |
| Y2124R | AACCATCCCATTTGTTTTGC |
| Y2125R | GTCGCCGACTGCATAGAGTT |
| Y2127R | AGGACAAGATCCACGGTGAG |
| Y2129F | GGGGCACAGAGTGGATGTAG |
| Y2134R | GGGAATTCGATTCTCTTGCTT |
| Y2136F | AATCGCGTGAAAGCACTTCT |
| Y2137R | CGCTGAGAGTTCTGCAAGTG |
| Y2511F | CTCCTCCTCGAACCTTCCTT |
| Y2511R | TAGCTCTCGCCGTTCTGTTT |
| Y2513F | TCGGACATCCACTCATCACT |
| Y2519R | GCTGAGGAGTCTTGAAGCAGA |
| Y2526F | GGGAGCCCAATTTGTCTATCT |
| Y2528F | TAGCTCTCGCCGTTCTGTTT |
| Y2528R | CTCCTCCTCGAACCTTCCTT |
| Y2530F | CAAGGGGAAGAAGGAGAAGG |
| SR3F | CCCAACTCTTCGTCTATCTC |
| SR5F | GCAGCAGTTCCGTTCTTG |
| SR9F | CCATTGAAAACTGCACACAA |
| 1x-4R | AACTGTTTCAAAGACCCAA |
| 1x-4F | ACCTACGTCAAGATAACCC |
| 2-1R | GATTAGTTACAGTAGCAGCAT |
| 2-1F | TGGGAGGTTTATTTGGTTA |
| 3-2R | ATGAGTTCATGGCAAACGG |
| 3-2F | TGCGGAAGTATTCTGGGTC |
| 3-3R | GGATCTAGGTAGCCGAAGC |
| 7-1F | CTCCATTTAGACCGCAACA |
| 8-1R | AGTAGGATAATGGCTAGGAGG |
| 11x-1F | GAGGAATAGAAGCCAACCC |
| 11x-2R | CCGCAATGACAGAATAAGG |
| 11x-2F | ACGGCAAACTACTTCAACC |
| 11x-3R | TTGCTGTTTGTGATGGTTA |
| Y3F | TGTGAGCCATTGCTGTTAGC |
| Y28F | GCTGTTGCTCCACATCTCCT |
| Y46F | GCGCAAGCGTAGGAGTTTAC |
| Y46R | TTGAACGATGAAAGGGAAGG |
| Y63R | CTCACCACGTGGCACTTATG |
| Y66R | TCCAGTTCTTTTTGCCTTGG |
| Y68R | TGGGCATTTAGCCATTTACC |
| Y88R | TTAGGGTGTTCTTTGGGCAC |
| Y414F | TCTCTCAAAATCTCAAACCCAGA |
| Y414R | GCTTAGGGCAAACCACTGAA |
| Y420F | CAAGCGATTGTTTCTCATCC |
| Y420R | CGTCATCATAAACCAACGTGC |
| Y435F | GGAACAGAGGCAACTGAGGA |
| Y435R | TCGAAGGCACAGAGAAGGTT |
| Y445R | GGGTCAGAACTGGACAAGGA |
| Y458F | AGGACTTGTCCACGTGCTTC |
| Y493F | ATGGGGTCATCCTCCATTTT |
| Y493R | TTTCTCCATTTGCCTTCACC |
| Y538F | CTAAACCCTAAACACAATATCTCC |
| Y538R | CATTATAAGGTCCCCAATGTC |
| Y542R | GAAGAAGAAGAATCCGACAG |
| Y567R | ATGAAATGAAAGAAAAGAAGGGAGG |
| Y604F | GACAATCCAGGCAGTCAGAG |
| Y604R | CATACTAATTAGCCATTCTCACCC |
| Y614R | GATCCACAAGAAACTGAAACTAG |
| Y640F | GCTTAGATCTGGACTACCGAATCCT |
| Y648R | CTACCCCCATTTTTTGGATTCACC |
| Y657F | CAATCTGAAAAACCATAAGACC |
| Y657R | ACCATTGAAACACCATGTC |
| Y672F | GCCCGCCATCTTTGAGGATCCG |
| Y672R | GGCAAAACTTGACAATTTTCTCGGC |
| Y732F | ACCGGTCAGTCCTCATAATC |
| Y736R | GAGGGCCATAGTCACCGG |
| Y751F | AATGCCACGGAGTACAATTT |
| Y751R | TGCAAGAGTGGAAGTTGAAA |
| Y762R | ACATTCACATTGGCATTCAC |
| Y792R | GATCAGCTTCCTTGTTGCTT |
| Y817F | CTTACTTTCCAACCTCTTTC |
| Y817R | TCGCTTAATTCTTTGGATGC |
| Y837F | AACCAGCCAAATTTCATCAC |
| Y864F | GGATTAATTAGCCCCCACAT |
| Y864R | TCTTTTTCAGCTTGGGTTCT |
| Y1082F | CCTACCAGCCAAAAAGAAAA |
| Y1082R | TCAAGGGTTTCAAGTTCCAT |
| Y1221F | CATGCAAATCCATGCTAGAG |
| Y1223F | CGGGCGATAAAATTCAGA |
| Y1223R | TTGGCTTCAACAGCTACAAA |
| Y1237R | ATACCAAGTCCTGCCGATTA |
| Y1248F | AATGTCAGCTGCCTATTTCC |
| Y1248R | AAGACAGGCGATGTCATCTT |
| Y1302F | ACTCGGCGTATAATTTGGAA |
| Y1521F | TCAACCGAAACAAGCTAACA |
| Y1623F | TCAACTATCAGTCCAAT |
| Y1623R | AAAGAGACCCACAAG |
| Y1991F | AGGTGAAGTGTAGAAGTAG |
| Y2002R | ATCACTTCAGCTGGGGTTT |
| Y2014R | AATTCCAGGAGTGCAGAAAC |
| Y2029F | TGGTTCTCTACGGTTTCTCC |
| Y2029R | GTTGGCTACGGTTACAATCC |
| Y2038F | GAGACACGAACACAAAAACG |
| Y2038R | GCGGTGTGTTATACATCCAT |
| Y2096R | TTAGTCAAACCGAGCAGCTT |
| Y2104F | CAAGCATCACCGTTGATTAG |
| Y2108F | GGGGACTTCATCTGGTTCTA |
| Y2108R | CAGTAGGCCAAGTCTCTCGT |
| Y2126R | TAACGGCTGTAACCCTTCTC |
| Y2128F | TAGGTCGAGGGGATCGATAG |
| Y2128R | CTAACAGGGGTGACATAGGG |
| Y2154F | CCCTGCTTACTCATTCCTCA |
| Y2155F | GGGAATTTCGATGCAATAAC |
| Y2182R | CACCTCCAAGGTCTTGATTT |
| Y2195F | GAACCTTTCTCCAACTCGAA |
| Y2195R | CTCTCGAACCTTAGGGCATA |
| Y2200F | TCTCAAAATTTACACAGCATCC |
| Y2200R | GCCTCGTATATCTCCATTGC |
| Y2214F | TCAATACAAAGCAGTTGAAGTC |
| Y2232F | ACAGGGCTGTTGAGAGAGTT |
| Y2240F | GGAAGCCCCGTTTATTTTAT |
| Y2252R | TACCTGGGTATCCAACCATC |
| Y2274F | TCCTCGGATTATCAAAACCT |
| Y2300R | GCTGTCGAAGTGAAGGAAGT |
| Y2419R | CACCAAATTCAGGTTCACAA |
| Y2434R | TGCTTCCTGCTAAAACATTG |
| Y2439F | GGCAACACAACAAAGGACTA |
| Y2440F | TCTATCCTGGGATGGAACAT |
| Y2443F | CGTTGAGAAGGAAAGCCTAA |
| Y2443R | AGCCTGCTTCATGTTCTTTT |
| Y2513R | CCCCTCTACCGAAATTGTTA |
| Y2544F | GCTGTGCTGGATTTAGTCG |
| Y2544R | CCGGTGTAGTAGCTCAAAGG |
| Y2562R | AGGAAGTAGGGCTGGTTGTA |
| Y2579F | TTTTCTAAGGCCTGGATTTG |
| Y2579R | TCTCTTCGTCTGCTTCTCCT |
| Y2636R | TGCAAGTCGGTCAAGTTTTA |
| Y2698F | ACCGGTTCAAATCAATCAAC |
| Y2698R | GCCATCTTATGATTCCCACT |
| Y2717R | TTTTACACCGAAATGCGATA |
| Y2722R | TTGAAAGGAAGGTTTCAAGG |
| Y2801F | GACGGTAAAAGGTCAACAGG |
| Y2970F | TCGATCTGAACCCATACTCA |
| Y4335F | CATTCGGCACGAGATTGG |
| Y4335R | GATTTGGTCCCCACTTTGG |
| Total | 32 AFLP, 298 SSR |

32 AFLP and 298 SSR primer pair combinations were used to screen for polymorphic primer pairs in genetic linkage map construction. The 50 AFLP primer pairs anchored onto the map were screened from combinations of the 32 AFLP primers, while the 30 SSR primer pairs anchored onto the map were obtained from previous research [17-18], and the 573 RSAMPL primer pairs anchored onto the map were screened from combinations of the 32 AFLP and 298 SSR primers.
