# Supplementary material for: Genetic Analysis and QTL Mapping of Seed Coat Color in Sesame (Sesamum indicum L.)
Source: PLoS One. 2013 May 21;8(5):e63898. doi: 10.1371/journal.pone.0063898 (PMC3660586; doi:10.1371/journal.pone.0063898)
Supplement: Table S3 — Fitness tests of five candidate genetic models for seed coat color analysis. The number of significant parameters, correlated with the adaptation level of the models, varied from 0–13. The E-0 model with the least number of significant parameters (0) in the three replications was selected from the five candidate models as the optimal model and was used for seed coat color analysis. *indicated significance at p = 0.05. (DOC) [file pone.0063898.s005.doc]

**Table S3** **Fitness tests of five candidate genetic models for seed coat color analysis.**

| **Test replication** | **Genetic model** | **Population** | **Paremeter for goodness-of-fit** | | | | | **No. of significant parameter(s)** |
| --- | --- | --- | --- | --- | --- | --- | --- | --- |
|  |  |  | **U_1_^2^** | **U_2_^2^** | **U_3_^2^** | **nW^2^** | **Dn** |  |
| Ⅰ | E_0 | P_1_ | 0.007(0.9312) | 0.039(0.8438) | 0.206(0.6499) | 0.0586 | 0.1393(n=25,CD(0.05)=0.2836) | 0 |
|  |  | F_1_ | 0.001(0.9702) | 0.041(0.8400) | 0.440(0.5073) | 0.0484 | 0.1069(n=27,CD(0.05)=0.2720) |  |
|  |  | P_2_ | 0.151(0.6973) | 0.211(0.6459) | 0.110(0.7401) | 0.0639 | 0.1231(n=28,CD(0.05)=0.2667) |  |
|  |  | BC_1_ | 0.447(0.5036) | 0.533(0.4655) | 0.109(0.7419) | 0.152 | 0.0602(n=213,CD(0.05)=0.0936) |  |
|  |  | BC_2_ | 0.137(0.7114) | 0.404(0.5250) | 1.231(0.2672) | 0.1828 | 0.0844(n=189,CD(0.05)=0.0995) |  |
|  |  | F_2_ | 2.179(0.1399) | 2.806(0.0939) | 0.969(0.3250) | 0.2927 | 0.0738(n=307,CD(0.05)=0.0779) |  |
|  | E_1 | P_1_ | 0.053(0.8185) | 0.056(0.8137) | 0.003(0.9570) | 0.0615 | 0.1266(n=25,CD(0.05)=0.2836) | 7 |
|  |  | F_1_ | 0.140(0.7087) | 0.445(0.5045) | 1.495(0.2214) | 0.0854 | 0.1265(n=27,CD(0.05)=0.2720) |  |
|  |  | P_2_ | 0.163(0.6867) | 0.123(0.7255) | 0.025(0.8749) | 0.061 | 0.1230(n=28,CD(0.05)=0.2667) |  |
|  |  | BC_1_ | 0.252(0.6158) | 0.363(0.5471) | 0.216(0.6419) | 0.1655 | 0.0654(n=213,CD(0.05)=0.0936) |  |
|  |  | BC_2_ | 0.917(0.3384) | 2.684(0.1014) | 8.092(0.0044)* | 0.4495 | 0.1231(n=189,CD(0.05)=0.0995)* |  |
|  |  | F_2_ | 4.187(0.0407)* | 7.054(0.0079)* | 7.283(0.0070)* | 0.7142* | 0.1106(n=307,CD(0.05)=0.0779)* |  |
|  | E_2 | P_1_ | 3.672(0.0553) | 2.970(0.0848) | 0.278(0.5979) | 0.3908 | 0.2216(n=25,CD(0.05)=0.2836) | 13 |
|  |  | F_1_ | 0.015(0.9010) | 0.001(0.9706) | 0.112(0.7381) | 0.042 | 0.1018(n=27,CD(0.05)=0.2720) |  |
|  |  | P_2_ | 5.334(0.0209)* | 4.493(0.0340)* | 0.218(0.6408) | 0.6191* | 0.2704(n=28,CD(0.05)=0.2667)* |  |
|  |  | BC_1_ | 6.478(0.0109)* | 4.116(0.0425)* | 3.037(0.0814) | 0.8717* | 0.1447(n=213,CD(0.05)=0.0936)* |  |
|  |  | BC_2_ | 2.899(0.0886) | 1.730(0.1884) | 1.777(0.1825) | 0.4467 | 0.1109(n=189,CD(0.05)=0.0995)* |  |
|  |  | F_2_ | 4.327(0.0375)* | 4.341(0.0372)* | 0.077(0.7814) | 0.48* | 0.0830(n=307,CD(0.05)=0.0779)* |  |
|  | B_1 | P_1_ | 15.881(0.0001)* | 16.077(0.0001)* | 0.365(0.5457) | 1.6086* | 0.4049(n=25,CD(0.05)=0.2836)* | 6 |
|  |  | F_1_ | 2.970(0.0848) | 2.786(0.0951) | 0.000(0.9982) | 0.32 | 0.2302(n=27,CD(0.05)=0.2720) |  |
|  |  | P_2_ | 0.868(0.3514) | 1.331(0.2487) | 1.010(0.3149) | 0.1802 | 0.1707(n=28,CD(0.05)=0.2667) |  |
|  |  | BC_1_ | 4.025(0.0448) | 3.314(0.0687) | 0.238(0.6257) | 0.6427* | 0.1200(n=213,CD(0.05)=0.0936)* |  |
|  |  | BC_2_ | 0.058(0.8103) | 0.504(0.4778) | 3.647(0.0562) | 0.2438 | 0.0921(n=189,CD(0.05)=0.0995) |  |
|  |  | F_2_ | 0.548(0.4592) | 1.374(0.2411) | 3.321(0.0684) | 0.1975 | 0.0697(n=307,CD(0.05)=0.0779) |  |
|  | B_2 | P_1_ | 13.282(0.0003)* | 12.877(0.0003)* | 0.057(0.8113) | 1.3495* | 0.3755(n=25,CD(0.05)=0.2836)* | 9 |
|  |  | F_1_ | 3.608(0.0575) | 3.298(0.0693) | 0.008(0.9267) | 0.3815 | 0.2430(n=27,CD(0.05)=0.2720) |  |
|  |  | P_2_ | 1.959(0.1617) | 2.371(0.1236) | 0.546(0.4598) | 0.2939 | 0.2042(n=28,CD(0.05)=0.2667) |  |
|  |  | BC_1_ | 5.647(0.0175)* | 4.999(0.0254)* | 0.068(0.7942) | 0.755* | 0.1227(n=213,CD(0.05)=0.0936)* |  |
|  |  | BC_2_ | 0.066(0.7972) | 0.039(0.8436) | 3.183(0.0744) | 0.2211 | 0.0783(n=189,CD(0.05)=0.0995) |  |
|  |  | F_2_ | 3.456(0.0630) | 4.693(0.0303)* | 2.149(0.1427) | 0.4125 | 0.0692(n=307,CD(0.05)=0.0779) |  |
| Ⅱ | E_0 | P_1_ | 0.001(0.9806) | 0.017(0.8949) | 0.188(0.6642) | 0.0268 | 0.0790(n=28,CD(0.05)=0.2667) | 4 |
|  |  | F_1_ | 0.007(0.9320) | 0.104(0.7471) | 0.921(0.3372) | 0.051 | 0.1013(n=30,CD(0.05)=0.2570) |  |
|  |  | P_2_ | 0.056(0.8122) | 0.113(0.7369) | 0.180(0.6716) | 0.0415 | 0.0891(n=30,CD(0.05)=0.2570) |  |
|  |  | BC_1_ | 4.384(0.0363)* | 5.227(0.0222)* | 1.072(0.3005) | 0.6204* | 0.1113(n=192,CD(0.05)=0.0987)* |  |
|  |  | BC_2_ | 0.090(0.7641) | 0.327(0.5672) | 1.269(0.2599) | 0.3509 | 0.0996(n=186,CD(0.05)=0.1003) |  |
|  |  | F_2_ | 0.675(0.4112) | 1.133(0.2872) | 1.153(0.2828) | 0.1367 | 0.0540(n=289,CD(0.05)=0.0803) |  |
|  | E_1 | P_1_ | 0.001(0.9797) | 0.024(0.8763) | 0.520(0.4708) | 0.0344 | 0.0827(n=28,CD(0.05)=0.2667) | 9 |
|  |  | F_1_ | 0.188(0.6644) | 0.553(0.4569) | 1.679(0.1951) | 0.0854 | 0.1252(n=30,CD(0.05)=0.2570) |  |
|  |  | P_2_ | 0.033(0.8560) | 0.014(0.9065) | 0.054(0.8154) | 0.0273 | 0.0687(n=30,CD(0.05)=0.2570) |  |
|  |  | BC_1_ | 7.401(0.0065)* | 8.156(0.0043)* | 0.787(0.3751) | 0.9151* | 0.1232(n=192,CD(0.05)=0.0987)* |  |
|  |  | BC_2_ | 1.146(0.2844) | 2.796(0.0945) | 6.464(0.0110)* | 0.5856* | 0.1345(n=186,CD(0.05)=0.1003)* |  |
|  |  | F_2_ | 1.693(0.1932) | 3.774(0.0521) | 7.459(0.0063)* | 0.4453 | 0.0947(n=289,CD(0.05)=0.0803)* |  |
|  | E_2 | P_1_ | 4.173(0.0411)* | 4.424(0.0354)* | 0.252(0.6158) | 0.4185 | 0.2042(n=28,CD(0.05)=0.2667) | 8 |
|  |  | F_1_ | 0.467(0.4946) | 0.659(0.4171) | 0.361(0.5481) | 0.0864 | 0.1338(n=30,CD(0.05)=0.2570) |  |
|  |  | P_2_ | 2.602(0.1067) | 2.674(0.1020) | 0.086(0.7689) | 0.3197 | 0.1922(n=30,CD(0.05)=0.2570) |  |
|  |  | BC_1_ | 0.002(0.9632) | 0.245(0.6206) | 3.245(0.0717) | 0.2711 | 0.0935(n=192,CD(0.05)=0.0987) |  |
|  |  | BC_2_ | 3.509(0.0610) | 2.467(0.1163) | 0.946(0.3308) | 0.6498* | 0.1413(n=186,CD(0.05)=0.1003)* |  |
|  |  | F_2_ | 9.914(0.0016)* | 8.851(0.0029)* | 0.086(0.7687) | 1.0099* | 0.1020(n=289,CD(0.05)=0.0803)* |  |
|  | B_1 | P_1_ | 12.581(0.0004)* | 13.336(0.0003)* | 0.757(0.3842) | 1.2399* | 0.3158(n=28,CD(0.05)=0.2667)* | 5 |
|  |  | F_1_ | 0.230(0.6312) | 0.284(0.5943) | 0.074(0.7860) | 0.0484 | 0.1059(n=30,CD(0.05)=0.2570) |  |
|  |  | P_2_ | 0.468(0.4937) | 1.028(0.3107) | 1.972(0.1602) | 0.1344 | 0.1349(n=30,CD(0.05)=0.2570) |  |
|  |  | BC_1_ | 0.000(0.9881) | 0.001(0.9788) | 0.002(0.9610) | 0.1487 | 0.0665(n=192,CD(0.05)=0.0987) |  |
|  |  | BC_2_ | 0.025(0.8752) | 0.253(0.6147) | 1.974(0.1600) | 0.3395 | 0.1004(n=186,CD(0.05)=0.1003)* |  |
|  |  | F_2_ | 1.305(0.2533) | 1.848(0.1741) | 1.025(0.3112) | 0.205 | 0.0600(n=289,CD(0.05)=0.0803) |  |
|  | B_2 | P_1_ | 13.249(0.0003)* | 14.153(0.0002)* | 0.904(0.3417) | 1.3053* | 0.3237(n=28,CD(0.05)=0.2667)* | 9 |
|  |  | F_1_ | 0.678(0.4104) | 0.737(0.3907) | 0.060(0.8063) | 0.0888 | 0.1270(n=30,CD(0.05)=0.2570) |  |
|  |  | P_2_ | 1.129(0.2880) | 1.813(0.1782) | 1.614(0.2040) | 0.2029 | 0.1606(n=30,CD(0.05)=0.2570) |  |
|  |  | BC_1_ | 0.033(0.8560) | 0.094(0.7589) | 0.276(0.5996) | 0.2052 | 0.0714(n=192,CD(0.05)=0.0987) |  |
|  |  | BC_2_ | 0.289(0.5910) | 0.021(0.8839) | 2.241(0.1344) | 0.3657 | 0.1021(n=186,CD(0.05)=0.1003)* |  |
|  |  | F_2_ | 7.312(0.0068)* | 8.395(0.0038)* | 1.247(0.2641) | 0.7647* | 0.0834(n=289,CD(0.05)=0.0803)* |  |
| Ⅲ | E_0 | P_1_ | 0.098(0.7544) | 0.078(0.7806) | 0.009(0.9224) | 0.0741 | 0.1218(n=30,CD(0.05)=0.2570) | 0 |
|  |  | F_1_ | 0.043(0.8357) | 0.021(0.8849) | 0.050(0.8227) | 0.0348 | 0.0971(n=29,CD(0.05)=0.2617) |  |
|  |  | P_2_ | 0.106(0.7448) | 0.124(0.7243) | 0.023(0.8807) | 0.0981 | 0.1497(n=25,CD(0.05)=0.2836) |  |
|  |  | BC_1_ | 2.421(0.1197) | 1.483(0.2233) | 1.335(0.2479) | 0.3677 | 0.1057(n=155,CD(0.05)=0.1099) |  |
|  |  | BC_2_ | 0.137(0.7115) | 0.309(0.5784) | 0.625(0.4292) | 0.3325 | 0.0907(n=180,CD(0.05)=0.1019) |  |
|  |  | F_2_ | 2.000(0.1573) | 2.745(0.0976) | 1.323(0.2500) | 0.3115 | 0.0651(n=323,CD(0.05)=0.0759) |  |
|  | E_1 | P_1_ | 0.541(0.4621) | 0.594(0.4410) | 0.055(0.8146) | 0.0831 | 0.1178(n=30,CD(0.05)=0.2570) | 5 |
|  |  | F_1_ | 1.099(0.2945) | 1.326(0.2495) | 0.298(0.5852) | 0.1215 | 0.1321(n=29,CD(0.05)=0.2617) |  |
|  |  | P_2_ | 0.019(0.8890) | 0.011(0.9173) | 0.016(0.9004) | 0.0853 | 0.1344(n=25,CD(0.05)=0.2836) |  |
|  |  | BC_1_ | 0.277(0.5985) | 0.004(0.9471) | 3.147(0.0761) | 0.2844 | 0.0964(n=155,CD(0.05)=0.1099) |  |
|  |  | BC_2_ | 0.372(0.5421) | 1.151(0.2833) | 3.727(0.0535) | 0.448 | 0.1078(n=180,CD(0.05)=0.1019)* |  |
|  |  | F_2_ | 2.811(0.0936) | 5.115(0.0237)* | 6.520(0.0107)* | 0.5332* | 0.0996(n=323,CD(0.05)=0.0759)* |  |
|  | E_2 | P_1_ | 1.825(0.1767) | 1.515(0.2184) | 0.096(0.7567) | 0.1896 | 0.1704(n=30,CD(0.05)=0.2570) | 6 |
|  |  | F_1_ | 0.206(0.6500) | 0.195(0.6585) | 0.000(0.9916) | 0.0411 | 0.1036(n=29,CD(0.05)=0.2617) |  |
|  |  | P_2_ | 1.915(0.1664) | 1.647(0.1994) | 0.051(0.8208) | 0.2996 | 0.2341(n=25,CD(0.05)=0.2836) |  |
|  |  | BC_1_ | 4.774(0.0289)* | 3.862(0.0494)* | 0.362(0.5475) | 0.7402* | 0.1533(n=155,CD(0.05)=0.1099)* |  |
|  |  | BC_2_ | 0.614(0.4333) | 0.249(0.6178) | 1.078(0.2991) | 0.3723 | 0.1101(n=180,CD(0.05)=0.1019)* |  |
|  |  | F_2_ | 2.862(0.0907) | 2.247(0.1339) | 0.309(0.5781) | 0.3997 | 0.0891(n=323,CD(0.05)=0.0759)* |  |
|  | B_1 | P_1_ | 8.863(0.0029)* | 8.289(0.0040)* | 0.000(0.9888) | 0.8449* | 0.2871(n=30,CD(0.05)=0.2570)* | 8 |
|  |  | F_1_ | 2.203(0.1377) | 1.859(0.1727) | 0.087(0.7678) | 0.2778 | 0.1780(n=29,CD(0.05)=0.2617) |  |
|  |  | P_2_ | 0.108(0.7422) | 0.209(0.6476) | 0.307(0.5795) | 0.1024 | 0.1512(n=25,CD(0.05)=0.2836) |  |
|  |  | BC_1_ | 8.658(0.0033)* | 10.119(0.0015)* | 1.763(0.1842) | 1.0385* | 0.1455(n=155,CD(0.05)=0.1099)* |  |
|  |  | BC_2_ | 0.289(0.5906) | 0.380(0.5376) | 0.146(0.7023) | 0.3325 | 0.0917(n=180,CD(0.05)=0.1019) |  |
|  |  | F_2_ | 2.232(0.1352) | 2.808(0.0938) | 0.842(0.3588) | 0.3045 | 0.0719(n=323,CD(0.05)=0.0759) |  |
|  | B_2 | P_1_ | 6.952(0.0084)* | 6.286(0.0122)* | 0.034(0.8546) | 0.6636* | 0.2626(n=30,CD(0.05)=0.2570)* | 9 |
|  |  | F_1_ | 2.962(0.0852) | 2.421(0.1197) | 0.195(0.6589) | 0.3579 | 0.1963(n=29,CD(0.05)=0.2617) |  |
|  |  | P_2_ | 0.135(0.7130) | 0.239(0.6253) | 0.280(0.5969) | 0.1057 | 0.1541(n=25,CD(0.05)=0.2836) |  |
|  |  | BC_1_ | 5.159(0.0231)* | 6.063(0.0138)* | 1.107(0.2926) | 0.7763* | 0.1405(n=155,CD(0.05)=0.1099)* |  |
|  |  | BC_2_ | 0.403(0.5256) | 0.670(0.4131) | 0.665(0.4149) | 0.361 | 0.0996(n=180,CD(0.05)=0.1019) |  |
|  |  | F_2_ | 2.777(0.0956) | 3.362(0.0667) | 0.775(0.3787) | 0.3775 | 0.0798(n=323,CD(0.05)=0.0759)* |  |

The number of significant parameters, correlated with the adaptation level of the models, varied from 0-13. The E-0 model with the least number of significant parameters (0) in the three replications was selected from the five candidate models as the optimal model and was used for seed coat color analysis. *indicated significance at p = 0.05.
